# Overweight and lifestyle behaviors of low socioeconomic elementary school children in Buenos Aires

**DOI:** 10.1186/1471-2431-9-17

**Published:** 2009-02-24

**Authors:** Valeria Hirschler, Karina Buzzano, Anabella Erviti, Noemi Ismael, Silvina Silva, Ricardo Dalamon

**Affiliations:** 1 Nutrition and Diabetes Department, Hospital Durand, Buenos Aires, Argentina; 2 Division of Pediatrics, Hospital Durand, Buenos Aires, Argentina

## Abstract

**Background:**

There is growing interest in understanding the role that lifestyle behaviors play in relation to children's weight status. The objective of the study was to determine the association between children s BMI and dietary practices and maternal BMI.

**Methods:**

330 students (168M) aged 8.9 + 2 y from 4 suburban Buenos Aires elementary schools, and their mothers aged 36.2 + 7 y were examined between April and September 2007. Mothers were asked about their children s lifestyle. Data included parental education levels socioeconomic status, mothers and children s BMI, and Tanner stage.

**Results:**

All families were in the low socio-economic class. 79% of parents had an elementary education or less. 61 (18.5%) of children were obese (OB) (BMI>95%ile per CDC norms), and 53 (16.1%) overweight (OW) (BMI>85<95%ile). 103 (31.2%) of mothers were OB (BMI>30 kg/m2), and102 (30.9%) OW (BMI>25<30). 63% the children were pre-pubertal. 40% had a TV set in their bedroom. 13% of the children skipped breakfast and only 38% watched TV ≤2 hours daily, as recommended. Multiple logistic regression analysis showed a positive association between children s OW/OB and drinking sweetened beverages (OR = 1.24; 95% CI, 1.02–1.52), TV viewing (OR = 1.30; 95% CI,1.05–1.62), and maternal BMI (OR: 1.07; 95% CI,1.02–1.12), and a negative association with eating breakfast (OR = 0.43; 95% CI, 0.19–0.97) adjusted for fruit and vegetables consumption, milk consumption, maternal educational level and socioeconomic class.

**Conclusion:**

Our results suggest that TV viewing, drinking sweet beverages, skipping breakfast, and maternal BMI are important predictive variables for childhood OW/OB.

## Background

The rise in the prevalence of overweight and obesity (OW/OB) in children is one of the most alarming public health issues facing the world today. Child OB and its consequences threaten the health of today's children to such an extent that they may, have a shorter lifespan than their parents [1]. Although the causes of OB in children are multifaceted, the recent upsurge in pediatric OB could be explained by changes in the availability of high caloric 'fast' food, pre-prepared meals, and soft drinks, an increase in time spent watching television, playing video games and using the internet, and a decrease in the opportunities for physical activity in schools and communities [2]. Mothers are in a key position to shape the environments of children, and there is increasing interest in the contribution of parenting behaviors to OB risk [3].

Although dietary habits and leisure-time activities have been implicated in OW/OB, no epidemiological studies, as far as we know, have examined the associations between these lifestyle habits and OB in Argentine children.

The objective of this study was to determine the association between children's body mass index (BMI) and lifestyle behaviors and maternal BMI in a group of Argentinean children from a low socioeconomic class.

## Methods

A cross-sectional survey included elementary school students and their mothers from four suburban schools in Buenos Aires between April and September 2007. The schools were randomly selected from two poor neighborhoods of the suburbs of Buenos Aires. The reason why a higher number of schools were not included was due to economic limitations. We calculated the sample size based on the prevalence of OW/OB in children reported in other elementary school studies in Buenos Aires [4]. Given the fact that the prevalence of OW/OB was approximately 33% among children in a similar population [4], the sample size was estimate to achieve that percentage with an error lower than 0.05. The sample size resulting with this error was 344 children, figure close to the sample size used.

The sampling design was a two-stage probability sample, the first stage selection of the schools, and the second stage an invitation to all of the students and their mothers to undergo testing. The overall response rate was 96.3% of mothers and students. Only one child per mother was included in the analysis. In cases of mothers with more than one child, one was randomly selected for the study, which excluded108 children. In addition, 3 more children were not analyzed, because they either did not have a living mother or were adopted. Exclusion criteria also included: missing BMI information, the informed consent not being signed, and self-reported pregnancy at the time of the examination. There was not a significant difference in the mean age (p = 0.68), BMI (p = 0.79), sex (p = 0.66), and socioeconomic class among all the school children and those who were included. The social worker report showed that all of the children in these neighborhoods attended elementary school.

All subjects were examined by the same physician. The study was approved by the Human Rights Committee of Durand Hospital in Buenos Aires. Each parent gave written informed consent and children gave assent after an explanation of the study and before its initiation.

Although Argentina is a Spanish-speaking country, the population differs greatly from what is usually referred to as Hispanic in the U.S. About 85% of the population is of European descent (largely Spanish and Italian), with the remainder of mixed European and Native American (12%) or Native American (3%) descent [5].

Socio-economic characteristics included age, parental level of education and the presence or absence of a refrigerator or a dirt floor. Mothers were asked to define their level of formal education as having either: no formal education, only the first three years of elementary school, completed elementary school, completed high school, or having a university degree.

Because many mothers would not have been able to understood the written questions, questionnaires about their children's lifestyle behaviors were all completed by a single pediatrician (VH). The questionnaires were pre-examined by a statistician and a psychologist, and were validated by administering twice in two weeks to a pilot group of 30 mothers. A high level of concordance was observed between test and re-test results. To assess physical activity, a four-level index that ranked participants according to the number of blocks walked daily was used (≤5, 5–10, 10–20, ≥20 blocks daily). A five-level index that ranked participants according to daily consumption of vegetables or fresh fruit, milk, and sweetened beverages, and number of hours of TV viewing was also done. Milk included any type of cows milk and then was subcategorized by percentage of milk fat. Sweetened drinks included any sweetened fruit juice, fruit-flavored drink (natural or artificial), or drink that contained fruit juice in part. Mothers were also asked whether their child ate breakfast or not. Standard serving sizes and food models were provided as a reference for intake estimation.

Height and weight were measured with subjects wearing light clothing and without shoes. Height was recorded to the nearest 0.1 cm with a wall-mounted stadiometer. Weight was measured to the nearest 0.1 kg on a medical balance scale. BMI was calculated as weight in kilograms divided by height in meters squared. The child's BMI percentile for age and gender were determined from Centers for Disease Control and Prevention data and used to identify children's status as normal weight (< 85^th ^percentile), OW (85^th ^to < 95^th^), or OB (> or = 95^th^) [6]. The physical examination included determination of the stage of puberty according to the criteria of Tanner1 [7] Pubic hair was staged from I, representing immaturity, to V, for full maturity [8].

### Data analysis

Chi squared test was used to compare proportions. When more than 20% of the cells had expected frequencies <5, Fisher's exact test was used. BMI was converted to age- and sex-standardized percentiles based on the Centers for Disease Control and Prevention 2000 growth charts, which are not race specific[8]. BMI z-score (BMI-z) and height z scores were also determined as CDC charts [8]. The fit to normal distribution of continuous variables was assessed using the Shapiro-Wilks test. The primary focus of the analysis was to determine if lifestyle behaviors and maternal BMI were associated with OW/OB in their children. Multiple binary logistic regression analyses was used to examine the association between independent risk factors and children's OW/OB as the dependent variable and observed associations was expressed as OR with 95% confidence intervals. P values ≤ 0.05 were considered statistically significant. Data were presented as mean ± SD. and observed prevalence are expressed as percentages. Analyses were done using the SPSS (Chicago, IL) statistical software package SPSS version 10.0 ^®^.

## Results

Three hundred and thirty students (168 Males) aged 8.9 ± 2 years from four suburban elementary schools, and their mothers aged 36.2 ± 7 years were examined between April and September, 2007. Participants came from a low socio-economic class. The educational backgrounds of the mothers were as follows: 4.5% (N = 15) had no formal education, 20% (N = 66) had only the first three years of elementary school, 53% (N = 175) had completed elementary school and 20.9% (N = 170) had some high school. Approximately 9% of the families did not have a refrigerator and 4.2% had a dirt floor.

There was a significantly lower mean z score for height in normal weight -0.53 ± 0.96 vs OW/OB children 0.08 ± 0.87 (p < 0.001). The probability of being OW/OB among tall (z-height ≥2) boys and girls was significantly greater than that for normal height children OR, 2.18 (1.01 to 4.70; p = 0.04). There was not a significantly association between stunting and OW/OB (OR,0.70 (0.18 to 2.70; p = 0.60) Tallness was significantly associated with OW/OB children, while stunting was not associated with OW/OB in the sample.

The sample sizes and BMI values from the children by age and gender are shown in Table 1. There was not a significant difference in the mean BMI percentile between boys and girls (p = 0.28). The prevalence of childhood OW was 16.1% (53/330), OB 18.5% (61/330) and underweight was 1.5% (5/330) with mean BMI z-scores for the OB of 2.04 ± 0.37; OW of 1.30 ± 0.14, and underweight -2.97 ± 1.18. There was not a significant difference in the prevalence of OW [15.5% vs. 16.7%] and OB [17.9% vs. 19.1%] between boys and girls (p = 0.89). Sixty three percent, 22.0%, 11.5%, and 2.6% were Tanner stage I, II, III, and IV. None was at Tanner stage V. The prevalence of Tanner stage I was significantly lower in girls (35.5%) than in boys (64.5%) (p < 0.001), as expected with earlier pubertal maturation in girls. There was not a significant difference in the z-BMI of the children according to Tanner stage.

**Table 1:** Sample Sizes and Descriptive Characteristics from the Children According to Gender and Age

Age	BMI	z-BMI	BMI Percentile	N
Boys				
6.00	17.37 ± 2.93	0.75 ± 1.13	67.76 ± 25.48	30
7.00	16.70 ± 2.54	0.51 ± .91	63.68 ± 25.62	38
8.00	17.63 ± 2.35	0.48 ± 1.31	66.67 ± 29.24	35
9.00	19.57 ± 3.15	0.76 ± .92	71.25 ± 26.23	19
10.00	20.01 ± 4.03	0.87 ± 1.08	72.79 ± 27.06	21
11.00	20.85 ± 6.16	0.33 ± 1.31	59.42 ± 34.76	14
12.00	19.32 ± 2.78	0.31 ± .85	59.86 ± 25.97	11

All Boys	18.39 ± 3.71	0.61 ± 1.08	66.88 ± 27.11	168

Girls				
6.00	16.69 ± 2.66	0.51 ± 1.08	64.81 ± 29.32	33
7.00	17.60 ± 2.64	0.58 ± .89	67.9 ± 24.29	24
8.00	18.66 ± 2.92	0.69 ± .99	68.75 ± 28.42	27
9.00	19.93 ± 3.30	0.87 ± .94	74.87 ± 26.05	23
10.00	18.80 ± 4.15	0.22 ± 1.41	58.87 ± 32.42	22
11.00	20.72 ± 3.71	0.78 ± .93	73.31 ± 26.33	15
12.00	20.46 ± 3.19	0.48 ± .78	65.63 ± 26.59	18

All Girls	18.82 ± 3.60	0.61 ± 1.05	67.99 ± 27.98	162

All Children	18.60 ± 3.66	0.61 ± 1.07	67.42 ± 27.50	330

Data are means ± Standard Deviation

The average maternal BMI was 28.11 ± 6.18 which indicates that, as a group, they were OW. Thirty one % (103/330) of the mothers were OB (BMI >30) and 29.7% (102/330) OW (BMI >25<30). The mean BMI of OW mothers was 27.42 ± 1.43 and of OB mothers, 35.71 ± 4.85. The prevalence of OW/OB was more common (p = 0.038) in mothers with a lower level of education (divided into elementary school or less and more).

There was a higher prevalence of OW/OB in mothers of the OW/OB children than for the normal weight children (74.6% vs 54.7%; p < 0.001). Children of mothers with OW/OB had mean values of z-BMI which were significantly higher than those for the children of mothers without OW/OB; z-BMI (0.85 ± 0.94 vs 0.25 ± 1.19; p < 0.001).

The reported median daily intake of sweet beverages was 4 glasses/day (interquartile range: 2–5), the median intake of fruit and vegetables 2 servings/day (interquartile range: 1–2), the median daily intake of milk was 2 servings/day (interquartile range: 1–2). The number of blocks walked daily 10 (interquartile range: 5–15) and the hours of TV watched 3/day (interquartile range: 2–4).

The reported frequency of children's lifestyle behaviors according to the presence or absence of OW/OB are presented in Table 2. Only 2.1%(7/329) children ate 5 or more fruit and vegetables servings by day. Only 1.8% drank low-fat or skim milk, as recommended for children who are older than 2 years [9].

**Table 2:** Lifestyle behaviors according to OW/OB

	Normal Weight	OW/OB	P value
TV ≤2 hs/d	76.6% (95/124)	23.4%(29/124)	0.001*
Non-TV in bedroom	71.9% (146/203)	28.1% 57/203)	0.004*
Skipping Breakfast	42.9%(18/42)	57.1%(24/42)	0.001*
Milk ≥3 glasses/d	78.8%(52/66)	21.2% (14/66)	0.008*
Sweet beverages >1 glass/d	65.1% (188/289)	34.9%(101/289)	0.37
Fruit and Vegetables≥5/d	85.7%(6/7)	14.3%(1/7)	0.24
Blocks ≤ 5/d	66.0% (92/139)	34.0% (47/139)	0.53
Mothers ≤ elementary school	65.9% (205/311)	34.1% (106/311)	0.34

Overweight and obesity were classified using the CDC cut-points

P-values compare percentages of lifestyle behaviors between Normal weight and OW/OB children. Chi squared test was used to compare proportions. Data are percentages. Significance *p < 0.05

The bivariate logistic regression analyses examining the association among dietary and sedentary habits and maternal BMI in children with OW/OB, showed that there was a negative relationship between breakfast intake and OW/OB (OR = 0.34; 95% CI, 0.17–0.66; p = 0.02). There was a positive relationship for sweet drink consumption (OR = 1.17; 95% CI, 1.003–1.37; p = 0.046), TV viewing (OR = 1.41; 95% CI, 1.18–1.68; p ≤ 0.001), and maternal BMI (OR = 1.09; 95% CI, 1.05–1.13; p ≤ 0.001). No significant association was found between OW/OB and fruit & vegetables, milk consumption, and blocks walked. This study also showed that children watching >2 hours of TV a day were significantly more likely to be higher consumers of sweet drinks (OR = 1.64 p = 0.050) advertised on TV.

Multiple logistic regression analysis showed a positive association between children's OW/OB and drinking sweetened beverages (OR 1.24;95% CI 1.02–1.52), TV viewing (OR 1.30; 95% CI 1.05–1.62), and maternal BMI(OR 1.07;95% CI 1.02–1.12), and a negative association with eating breakfast (OR 0.43;95% CI 0.19–0.97) adjusted for fruit and vegetables consumption, milk consumption, maternal educational level and socioeconomic class (Table 3).

**Table 3:** Association between lifestyle behaviors and children's OW/OB

Variables	Sig.	OR	95.0% C.I. for OR	
			
			Lower	Upper
Sweet beverages	0.033*	1.24	1.02	1.52
Fruits & vegetables	0.543	0.92	0.69	1.21
Milk	0.161	0.82	0.63	1.08
Breakfast	0.043*	0.43	0.19	0.97
TV viewing	0.016*	1.30	1.05	1.62
Maternal BMI	0.004*	1.07	1.02	1.12
Maternal education	0.158	1.27	0.91	1.78
Socioeconomic class	0.066	3.08	0.92	10.23

OW/OB were classified using the CDC cut-points

Multiple Logistic Regression Analysis. Dependent variable: children's OW/OB. Significance* p < 0.05

## Discussion

The 2003–2004 US National Health and Nutrition Examination Survey of 3958 children and adolescents aged 2 to 19 years found that 33.6% of children had a ≥85th percentile BMI [10]. The prevalence of OW/OB in this cohort of Argentinean children aged 5 to 13 years (34.6%) was very similar to the alarming and increasing rate of OB among children in the United States. Different studies have documented the increasing prevalence of OW/OB among adults and children in many countries throughout the world [11-13]. Similar results were found in a recent school study in Buenos Aires, Argentina [4]. Only 1.5% of the children were underweight. Even if low income populations in South America showed an association between stunting and OW/OB in children [14], this study did not find this association. This finding could be due to their lifestyles and also because meat is very cheap in Buenos Aires and even low socioeconomic class can eat meat.

In this sample, OW/OB in children was associated with skipping breakfast, increased television viewing time, drinking sweet beverages, and maternal BMI. These observations highlight the importance that lifestyle behaviors play in the childhood obesity epidemic. The reported intake of healthy foods was extremely low in this survey.

Fruits and vegetables have been promoted for the prevention of childhood OB because of their low energy density, high fiber content, and satiety value. Two studies reported that fruit consumption was associated inversely with weight status in children, [15,16] but a relationship with vegetable intake was not apparent. Consistent with this survey, several studies found no association between fruit and vegetable intake and childhood OB [17-20]. Only 2.1% of the children in this study ate 5 or more portions of fruits and vegetables by day. This could be due to sample size, or some vegetables typically were consumed with fat added during preparation, or just reflect the low consumption in our sample.

At the same time, most of the children reported drinking sweet beverages more than once per day. Consumption of sugar-sweetened beverages may be a key contributor to the epidemic of OW/OB, because these beverages have high added sugar content, low satiety, and incomplete compensation of total energy [21]. We found as well, in the regression analysis a significant association between sweet drinks consumption and OW/OB children.

Several studies have shown that skipping breakfast decreased the nutritional quality of the diets of children [22,23]. Evidence supports the view that OB children are more likely to skip breakfast than their leaner counterparts [15]. OB children also have been reported to eat smaller breakfasts than their non-obese peers [24]. We did observe, as well, a significant association among skipping breakfast habits, and OW/OB. The strength of the evidence is somewhat limited, however, because what constitutes a normal breakfast has not been defined consistently [24].

In the present study we observed high prevalence rates of sedentary behaviors and there was a strong trend for increased OW/OB with increased TV viewing time. These results support the growing body of evidence implicating television viewing as a major factor in childhood OB [25-28]. Several mechanisms have been suggested that link television viewing with OW/OB children. These include an increased dietary intake from eating during viewing or from the effects of food advertising, decreased energy expenditure during viewing, and reduced energy expenditure from television viewing displacing physical activity. [29-31]. Watching TV during meals is associated with increased frequency of poor food choices and decreased frequency of good choices [31,32]. Consistent with these studies, we found that children watching more than two hours of TV a day were significantly more likely to be higher consumers of sweet drinks.

OB is commonly familial [33,34]. Parental OB greatly increases the risk of a child becoming obese [35]. An OW school-aged child with an obese parent has over a 70% chance of being OB in young adulthood. [36]. Consistent with these studies, there was a higher prevalence of OW/OB in children with OW/OB mothers. Multiple regression analysis also showed that mothers' BMI was associated with their child's OW/OB. These findings suggest that mother's OW/OB plays a critical role in their children's weight.

Several limitations of this study should be acknowledged. First, it was a cross sectional study and therefore, we were unable to draw conclusions about causation and make observations over time. Nonetheless, appropriate analysis of cross-sectional data is a valuable initial step in identifying associations between behaviors and OW/OB. Second, the use of the information about calorie intake was not used because it was considered unreliable due to the low parental educational level. Despite these limitations inherent to using reported behavioral data, we still found associations among OW/OB and lifestyle behaviors that were consistent, strong, and in the expected direction. Third, the sample was not randomly selected; however, measures of BMI and waist circumference were comparable to those reported in other elementary school studies in Buenos Aires [4]. Fourth; the reason why a higher number of schools were not included was due to economic limitations. Lastly; as the prevalence of obesity was higher than 10%, OR by logistic regression analysis could overestimate the risk.

The strengths of our study included our school sample, which was more likely to represent the general population of school children, the good response rate of the children, and the use of regression models and simultaneous adjustments of confounding variables, such as physical activity and the intake of other food groups, in the association of lifestyle behaviors with OW/OB.

## Conclusion

Our results suggest that maternal OB, watching TV, skipping breakfast, and sugar-sweetened beverage consumption are important predictive variables for childhood OW/OB. Most of these risk factors can be modified, suggesting opportunities for preventive intervention during childhood to avoid the development of OW/OB.

## Abbreviations

(OB): Obesity; (OW): Overweight.

## Competing interests

The authors declare that they have no competing interests.

## Authors' contributions

VH conceived of the study, performed the statistical analysis and participated in its design and coordination KB participated in its design, AE, NI, SS and RD carried out the anthropometric measures and the questionnaires. All authors read and approved the final manuscript.

## Pre-publication history

The pre-publication history for this paper can be accessed here:


